# City consumption profile: a city perspective on the spending behavior of citizens

**DOI:** 10.1007/s41109-021-00406-2

**Published:** 2021-08-23

**Authors:** Alessia Galdeman, Cheick T. Ba, Matteo Zignani, Christian Quadri, Sabrina Gaito

**Affiliations:** Department of Computer Science, University of Milan, Milan, Italy

**Keywords:** Urban computing, Customer segmentation, Community detection

## Abstract

In designing the city of the future, city managers and urban planners are driven by specific citizens’ behaviors. In fact, economic and financial behaviors, and specifically, which goods and services citizens purchase and how they allocate their spending, are playing a central role in planning targeted services. In this context, cashless payments provide an invaluable data source to identify such spending behaviors. In this work, we propose a methodology to extract the consumption behaviors of a large sample of customers through credit card transaction data. The main outcome of the methodology is a concise representation of the economic behavior of people residing in a city, the so-called *city consumption profile*. We inferred the city consumption profile from a network-based representation of the similarity among the customers in terms of purchase allocation; on top of which we applied a community detection algorithm to identify the representative consumption profiles. By applying the above methodology to a set of credit card transactions of an Italian financial group, we showed that cities, even geographically close, exhibit different profiles which makes them unique. Specifically, usage patterns focused on a single type of good/service—mono-categorical consumption profile—are the main factors leading to the differences in the city profiles. Our analysis also showed that there is a group of consumption profiles common to all cities, made up by purchases of primary goods/services, such as food or clothing. In general, the city consumption profile represents a tool for understanding the economic behaviors of the citizens and for comparing different cities. Moreover, city planners and managers may use it in the outline of city services tailored to the citizens’ needs.

## Introduction

According to the United Nations report (https://unhabitat.org/sites/default/files/2020/10/wcr_2020_report.pdf), today 56% of the world’s population lives in urban areas, a proportion that is expected to increase to 68% by 2050, with 1.7 billion people around the globe moving into a city every week. Cities are and will be increasingly demanded to face a myriad of challenges, big and small, pressing and long-term, to manage the current urban environment and social fabric, and to build their desired future.

If the big general challenges are mostly common to all urban areas, designing effective solutions cannot be taken out of the city profile (Zygiaris 2013; Kitchin et al. 2015), as each urban area has distinct characteristics and qualities that all together make it unique. Existing literature on urban studies has started addressing the issue of creating the profile of the cities, but we are still a long way from being able to reconstruct comprehensive profiles, including the wealth of aspects and facets that make a city one of a kind (Moustaka et al. 2017, 2020).

In this context, urban science research (Lobo et al. 2020; Silva et al. 2019; Batty 2021) is seeking to provide tools and results for a fine comprehension of cities, increasingly within a citizen-centric approach. Thanks to world-wide data, it is nowadays possible to analyze several aspects of cities, such as wealth, health, economy, transportation and technology adoption (Ratti 2018; Calabrese et al. 2014; Xiaowen et al. 2020; Leo et al. 2018). For instance, the crime level of cities was explored by using socio-economic conditions, mobility information and physical characteristics of the neighborhood, however it has been proven that socio-ecological factors of neighborhoods relate to crime very differently from one city to another. Thus, it highlights that there is no “one fits all” city model (De Nadai et al. 2020) and citizens’ behavior are very heterogeneous across cities. Studies following a close approach have been also carried out on the happiness of citizens (Daniele et al. 2014; Alshamsi et al. 2015). A study closer to ours shows that the diversity of goods and services within a city neighborhood can define its economic growth (Chong et al. 2020). Finally, Di Clemente et al. (2018) have used a text compression technique on the sequences of credit card purchases, as here, to show that it is possible to detect ubiquitous patterns of collective behavior.

In a step toward achieving a city profile within a people-centric approach, in this work, we address a specific aspect of cities: the *city consumption profile*. The city consumption profile summarizes the consumption patterns of its citizens which depend on how people allocate their spending amount among the merchant categories and on the volume of their purchases. To achieve the goal, our methodology adopts two of the main driving paradigms in current scientific research. The first is a data-driven approach. As cashless payments continue to mature at a dramatically increasing rate also imposed by the COVID-19 pandemic, consumer digital footprints are becoming massive. While an immediate and obvious consequence is a definitive shift from traditional to digital marketing, here we leverage citizens’ digital footprints to shape their city consumption profile. The second is a modeling approach based on complex networks theory. We model the relations customers-to-merchant categories as a bipartite graph on which to build a similarity graph between customers based on their consumption preferences. Community detection algorithms enable us to perform customer segmentation and to find out groups of people with similar spending habits. Thus, the sum of citizens’ profiles, expressed as groups, outlines, shapes, and defines the profile of their city. Finally, we enable the city to explore its overall diversity from other ones, by providing a city consumption similarity index.

The methodological pipeline is applied to real-world case studies. We leverage a set of credit card transactions provided by one of the main Italian financial institutions as cashless payment data source and show the purchase profile of several cities. Results show that basic, ordinary purchases of everyday life are common to all cities, but that each city exhibits peculiar traits on specific aspects. Thus, cities, also geographically close, exhibit different profiles which make them unique, even for the city consumption profile only.

## Data

In this study, we consider the set of purchases made through credit cards by the customers of an Italian financial group, spanning a 3-year period, from February 2017 to May 2020. In terms of data volume, the dataset contains more than 130 million transactions made by more than 600.000 credit cards. Each transaction contains:

- the amount of the transaction, corresponding to the price of the purchased good/s;
- the anonymized ID of the credit card who has bought the good/s;
- the *Merchant Category Code (MCC)* of the legal personality selling the good/s. According to ISO 18245, the MCC is a four-digit number used to identify the type of business of a retailer/merchant based on the goods it provides. It expresses a two-level hierarchy, where the first two digits describe general merchant categories, while the latter ones stand for a more specific classification. For instance, the MCC 7311 represents *Advertising Services* and 73 represents in general *Business Services* (https://www.citibank.com/tts/solutions/commercial-cards/assets/docs/govt/Merchant-Category-Codes.pdf). MCCs are used for tax reporting, interchange promotion, and gathering information about cardholder’s purchasing behavior.

The identification and the segmentation of the spending behavior of customers rely on MCCs, since they provide a standard categorization of retailers and goods, common to the majority of the financial institutions. For the purpose of this study, the granularity of the merchant category is too fine. For instance, the merchant category 5411, standing for *Grocery stores, Supermarkets*, and 5462 for *Bakeries*, are very similar, because they are mainly related to the consumption of alimentary products. In these cases, we exploit the two-level hierarchy of merchant categories and take the *macro-categories* expressed by the first two digits (https://www.citibank.com/tts/solutions/commercial-cards/assets/docs/govt/Merchant-Category-Codes.pdf).

The data follow a general format: each card is fully described by a sequence of purchases, where each purchase is a pair: the amount spent and a categorical label related to the type of purchase. In our case, the sequence of purchases is extracted from transactions made through a cashless payment system, i.e. credit cards.

It is worth noting that no temporal information is needed as we are interested in getting a snapshot of the overall city behavior, not the single card behavioral time series. Moreover, the dataset provided was pseudo-anonymized. Specifically, card identifiers were replaced by a randomly generated ID.

## Methodology

In this section, we detail the process for obtaining the city consumption profile, starting from the credit card transactions made by citizens. Specifically, the entire process is divided into four parts:

1. client-centric graph-based data modeling: a weighted customer/merchant category bipartite graph is built from the records of credit card transactions;
2. customer consumption profile: each customer is described by how s/he allocates their purchases into the merchant categories;
3. customer segmentation: we identify groups with similar purchase profiles;
4. city consumption profile: we define the city consumption profile by assigning to the members of the previous groups their home location, and we compare them to assess whether cities and their citizens are similar in terms of spending amount and types of consumption.

In short, to set up a city consumption profile, we adopt a data-driven approach and a graph-based methodology. In the following, we describe the whole methodological pipeline, which are the only data needed to shape the city profile and we deepen the above four steps.

### Methodology pipeline

**Fig. 1:**
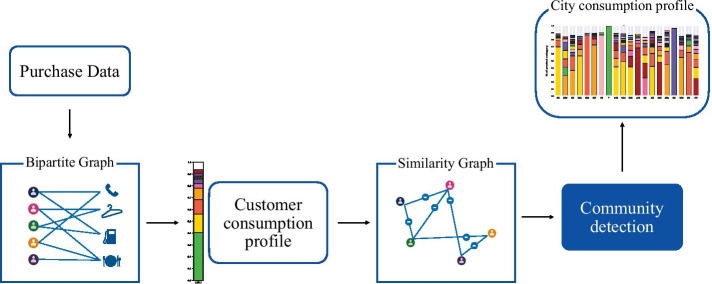
Methodology pipeline: A summary of the pipeline to extract and analyze the city consumption profiles. The transformation and analysis pipeline moves from left to right; from the raw purchase data, we build a bipartite graph (customer-to-merchant category). From the bipartite graph, we derive a customer consumption profile, which summarizes the single spending behavior of clients. The customer consumption profiles are nodes of a similarity graph where edges connect very similar consumption profiles. As the last step, a community detection algorithm, run on the similarity graph, identifies the representative consumption profiles used to obtain the city consumption profile

Given the data schema illustrated in the previous section, we adopt a graph-based methodology to identify and study the city consumption profiles. The pipeline of the methodology is depicted in Fig. 1.

In the first step, we represent the purchase data as a graph. Specifically, we chose a bipartite graph representation, where one class of nodes is made up of customers, and the other is made up of merchant categories. A link exists between a customer-node and a category-node if the former has purchased a good sold by a retail or service belonging to that merchant category, at least once. A link is weighted; the weight is defined as the amount of money spent by the customer on that category over the period of the dataset. In the second step—the customer representation layer—we map each customer into a vector space by assigning to each customer a feature vector—the *consumption profile*. The consumption profile captures how the client distributes her/his money available for purchasing among the merchant categories. The mapping into a vector space allows assessing the similarity of the customers’ spending behavior in terms of their distances. In the third step, we exploit such similarities to identify groups of customers with similar consumption profiles. The group identification is performed through a community detection algorithm performed on the similarity network of the customers. The outcome of this phase is a set of communities, which represent different ways of allocating the spending amount among the merchant categories, i.e. different representative consumption profiles. Finally, the city consumption profile is built on top of the representative consumption profiles and on their distribution among the customers residing in a city. In the remainder of the section, we detail each step of the pipeline.

### Bipartite graph

The first task is modeling the credit card transaction data into a graph representation that totally describes them. We model the purchase data by a bipartite graph since it well represents the relation between two types of nodes: the clients and the merchant categories associated with their purchases. Formally, we denote as *C* the set of clients and *M* the set of merchant categories, forming the node set of the graph. A link between a node $c\in C$ and a node $m\in M$ indicates that the customer *c* has bought at least one good sold in/by a retail belonging to the merchant category *m*. Moreover, we add a weight to each link (*c*, *m*) which corresponds to the total amount of money spent by *c* in retails or services belonging to the merchant category *m*. The link weight summarizes the purchase history of a client in a specific merchant category, while the links outgoing from *c* capture the distribution of *c*’s spending amount among the categories, i.e. the spending behavior of the client.

### Customer representation through consumption profile

A first raw representation of clients’ spending pattern can be obtained by gathering together in a vector all the outgoing weights from a client *c*. The resulting vector ${\mathbf {v}}^c$ has a dimension equal to the cardinality of *M* and each component is described as:

$$\begin{aligned} {\mathbf {v}}_i^c= {\left\{ \begin{array}{ll} w(c,i)=\sum _{j = 1}^{n}{p_j^i} &{} \quad \text {if } n>0 \\ 0 &{} \quad \text {otherwise} \end{array}\right. } \end{aligned}$$

where $p_J^i$ indicates the price of the good purchased by *c* in a shop/retail/service whose category is *i*; and *n* is the number of purchases in the category *i*.

The vector ${\mathbf {v}}^c$ should describe the spending pattern of a client, however, its formulation presents some limits for the purpose of the study. The first problem concerns the scale of the purchases. Since the variability of the spending amount, it is difficult to compare vectors with different orders of magnitude. Second, we observed that vectors have many non-zero elements, but most of them are marginal since their total amount is very low w.r.t. the main elements. To overcome the above limitations, we apply the following transformations:

- *Normalization* we normalize the vector ${\mathbf {v}}^c$, so that it represents a probability distribution over the merchant categories in *M*;
- *Tail cut* we cut the tail of the distribution, after normalization, by putting to zero the elements whose value did not contribute to reaching 99% of the total, i.e. we remove the categories below the first percentile of the distribution of the normalized components of each consumer vector. Finally, we normalize again the “cut” vector. From our viewpoint, this methodology has two advantages: (1) the choice of a low threshold on the percentile allows us not to lose discriminating information from the consumer vector. In fact, for each client, only their marginal categories are discarded: the median number of put-to-zero categories is 1; and (2) the tail cut is more targeted to each user w.r.t. other feature selection approaches. Indeed, the latter act on the whole dataset, discarding the same set of categories for all the customers. Consequently, groups of similar spending behaviors whose similarity depends on the discarded categories may be misidentified or not identified at all^1^.

**Fig. 2:**
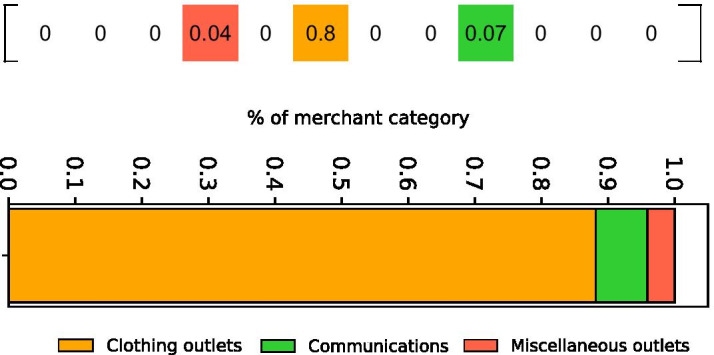
Visualization of a consumption profile. At the top the whole consumption profile with zero-elements. Then, the vector is displayed as an horizontal stacked barplot where each color segment corresponds to a specific merchant category and its width is proportional to the value in the consumption profile vector

We name the resulting vector as *consumption profile*. In the following, we visualize the consumption profile as a stacked barplot, as depicted in Fig. 2. The graphic depicts the 12-dimension vector [0, 0, 0, 0.04, 0, 0.88, 0, 0, 0.07, 0, 0, 0]; each bar represents a non-zero category and the bars are stacked in descending order according to the probability of the category. Thus, the customer consumption profile reports the distribution of the client’s spending amount among the merchant categories. Given the example above, that client employs most of her/his spending amount in essential goods, such as clothing and food.

### Customer segmentation

In the definition of the spending behavior of customers residing in a city we require a compact representation of the purchase patterns, so to increase the readability and the explainability of the results to non-expert decision makers. To this aim, we move towards the identification of representative patterns in the allocation of the spending amount, through the grouping of similar behaviors. Specifically, we adopt an approach based on the projection of the initial bipartite graph into one with a single type of node—the clients—which exploit the mapping of the latter into a vector space. For this purpose, we create a similarity graph on the client set, where we assign to a link (*u*, *v*) the similarity between *u*’s and *v*’s consumption profiles. As similarity measure we adopt the *cosine similarity*:

$$\begin{aligned} sim(x,y) = \frac{\sum _{i = 1}^{N}{x_{i} \cdot y_{i}}}{\sqrt{\sum _{i = 1}^{N}{x_{i}^{2}}}\cdot \sqrt{{\sum _{i = 1}^{N}{y_{i}^{2}}}}} \end{aligned}$$

The cosine similarity overcomes some limitations of other measures we considered in building the similarity graph. For example, the Jaccard similarity, despite its very low computational cost, is not suitable for a vector representation of the customers’ spending behavior since it is based on set operations. We have also considered the Euclidean similarity, more suitable for vector space models but with some drawbacks, e.g. the absence of minimum and maximum values and a high computational cost. In addition, the Euclidean similarity suffers from the curse of dimensionality but, in our case, its effects are not so evident due to the normalization step. Thus, the cosine similarity behaves better than the previous similarity measures: (1) it is fast to compute, if we measure in advance the norm of the vectors; (2) it performs better with sparse vectors because the resulting value is more influenced by common components; and (3) it has also a limited domain, i.e. the interval [0, 1]. From the fully connected graph of similarities, we prune all links but the strongest ties, obtaining a sparsification of the initial similarity graph. Specifically, we keep a link (*u*, *v*) if $sim(u,v)> \delta$.

Finally, we segment the consumption profiles associated with clients into groups with similar behavior. For the segmentation (Mihova and Pavlov 2018; Zakrzewska and Murlewski 2005; Zheng et al. 2014), we apply the Louvain algorithm (Blondel et al. 2008)—a graph-based community detection algorithm—on the pruned similarity graph. The Louvain algorithm assigns to each client a unique group—the *community*. The resulting communities contain customers that have similar behavior in spending and allocating their liquidity. Once the communities have been identified, we proceed with the final step: the computation of representative behaviors. Given a community matrix, i.e. a matrix whose rows are the consumption profile of clients in a specific community, the representative vector corresponds to the centroid of the community—the average over the columns of the community matrix. This way, each element of the representative vector is the average percentage of the spending amount that the customers in a community spent in a given merchant category.

### City consumption profile

After group segmentation and the identification of representative spending behaviors, customers are characterized by the representative consumption profile of the community they belong to. The population can also be partitioned into sub-populations to assess their diversity. Here we are interested in building city consumption profiles by conducting a research on the spending behavior of citizens of different cities to evaluate their common traits and diversity. To carry out this research, it is necessary to have geo-spatial information to identify the citizens of the urban area to be considered.

Given a city and its citizens, along with their representative consumption profiles, we define the *city consumption profile* by aggregating the representative consumption profile of its citizens and ranking the profiles according to their frequency among the citizens. Since the representative consumption profiles are common to all the cities, we can assess how much similarly the citizens of different cities behave. In particular, to assess the similarity among cities, we use a rank correlation approach, since the number of clients residing in the different cities is heterogeneous. For each city, we rank the community indexes by their cardinality. For instance, considering only three communities—*a*, *b* and *c*-, if a city has more citizens in the second community (*b*) and the smallest percentage in the first one (*a*), its rank vector will be [*b*, *c*, *a*]. In Table 1 we report how the rank vector is computed on data related to the city of Bergamo. The first column represent the index of the representative consumption profile in the graph, the second column lists the percentage of citizens having a specific representative profile, while the final column shows the rank vector, where each element is the profile column ordered by the values in the second column by descending order. Then, we apply the weighted Kendall’s $\tau$ to the city rank vectors.

**Table 1:** Ranked city consumption profile

Representative consumption profile	% of citizens in profile	Rank
0	4.4	0
1	2.0	1
2	1.3	2
3	1.2	4
4	1.2	5
5	1.0	7
6	0.6	11
7	0.5	14
8	1.0	6
9	1.2	3
10	0.6	12
11	0.8	9
12	0.8	8
13	0.5	15
14	0.7	10
15	0.5	16
16	0.2	19
17	0.4	18
18	0.5	17
19	0.6	13

In the table we report the rank associated to the city consumption profile of the city of Bergamo. The first column contains the identifiers associated to representative profiles. The second column reports the percentage of adoption of a specific representative profile over the citizens. In the third column we report the rank of the representative consumption profile. For example, the profile 5 characterized the $1\%$ of the Bergamo citizens and it is at the 7th position in the rank

Weighted Kendall’s $\tau$ is a coefficient that measures the similarity of two rankings; it is proportional to the number of pairwise adjacent swaps needed to convert one ranking into the other. Kendall’s $\tau$ has become a standard statistic to compare the correlation between two rank vectors, mostly due to its fast computation. On the other hand, Kendall’s $\tau$ presents some problems, solved by some weighted variants. The problems mainly regard the assignment of a low $\tau$ value for ranking with top elements that are quite identical: this is due to the introduction of noise by non-top elements that have a slightly different rank. We cope with this drawback by adopting a weighted version, defined in (Vigna 2015):

$$\begin{aligned} \tau _{w}(r,s) = \frac{\langle \mathbf{r}\mathbf , \mathbf{s}\mathbf \rangle _{w} }{\Vert \mathbf{r}\mathbf \Vert _{w} \cdot \Vert \mathbf{s}\mathbf \Vert _{w} } \end{aligned}$$

where $$\langle \mathbf{r}\mathbf , \mathbf{s}\mathbf \rangle _{w} = \sum _{i<j}{\text {sgn}(r_{i}-r_{j})\text {sgn}(s_{i}-s_{j})w(i,j) }$$ and $$\Vert \mathbf{r}\mathbf \Vert _{w} = \sqrt{\langle \mathbf{r}\mathbf , \mathbf{r}\mathbf \rangle _{w}}$$.

The standard non-negative symmetric weight function *w* is the *hyperbolic* function. The resulting correlation coefficient summarized how much the citizens in the two different cities behave alike in terms of distribution of spending amount among the merchant categories. We name the correlation coefficient as *city consumption similarity index*.

## Results

In this section, we report the results about the customers’ spending behaviors and the city consumption profiles of some of the most important cities in Northern Italy derived by applying the above methodology to the credit card transaction dataset (see “Data” section). Specifically, the results presented herein are based on a bipartite graph, with more than 12 million links, defined as follows:

- node set *C* contains all the customers who have purchased at least one good along the observation period—about 450K clients;
- node set *M* contains all the merchant categories (52) at the granularity of the macro-categories, i.e. the categories referred by the first two digits of the merchant code. Since merchant codes depend on the payment processor, we report the merchant coding provided by the financial group in the “Appendix” (see Fig. 7).^2^

Grouping the merchant categories into macro-categories is motivated by the excessive specialization offered by the four-digit product category. In fact, an experiment run with micro-categories on the same set of clients showed that customer segmentation results into different communities whose representative consumption profiles were very similar. For example, if a community contains clients who spend mainly in the micro-category 5621 (women clothing) and another community’s clients concentrate their purchases in the micro-category 5651 (general clothing), for the purposes of this study they should be merged since both communities spent mainly in clothing, so they have similar spending patterns.

**Fig. 3:**
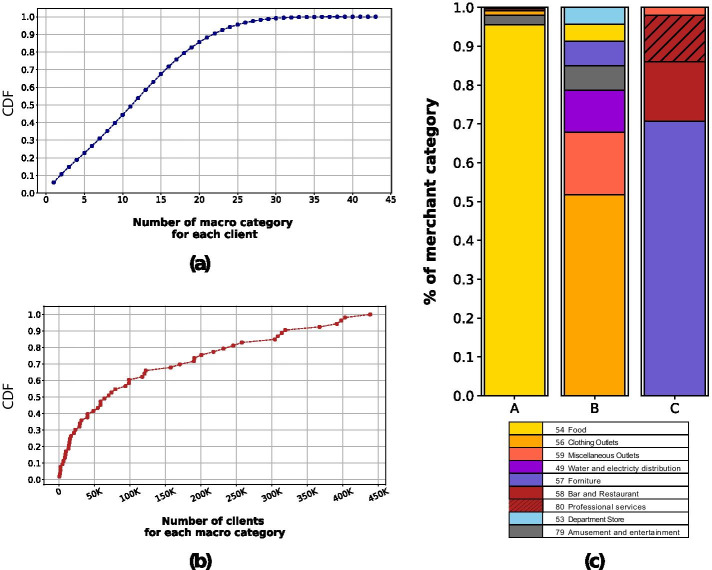
Properties of the bipartite graph and consumption profiles. In **a** the distribution of the number of different merchant categories per customer. In **b** the distribution of the number of customers associated to a merchant category. In **c** three examples of consumption profiles displayed as stacked barplots. In each stacked barplot, the height of the bar is proportional to the percentage of money spent for goods/services in the merchant categories indicated in the legend

By inspecting the degree distributions on both sets *C* and *M*, we gain some insights about the most favorite merchant macro-categories and on the diversity of the macro-categories customers spend money in. As for the latter variable, in Fig. 3a we report the cumulative distribution function (CDF) of the number of different macro-categories in which a customer has purchased goods or services. The distribution grows linearly up to 20 different categories—close to the 90th percentile-, indicating a quite uniform distribution on the number of different categories. The remaining 10% of the population spends in more than 20 different macro-categories with a small portion covering almost all the categories. In general, the spending behavior of most of the customers is focused on a small fraction of the available merchant categories. A similar trend also emerges from the analysis of the distribution of the number of clients per macro-category, as displayed in Fig. 3b. Here, we observe a limited set of macro-categories which are targets of a large fraction of the customers. This set contains merchant categories related to retailers selling primary goods, such as food or clothing.

**Fig. 4:**
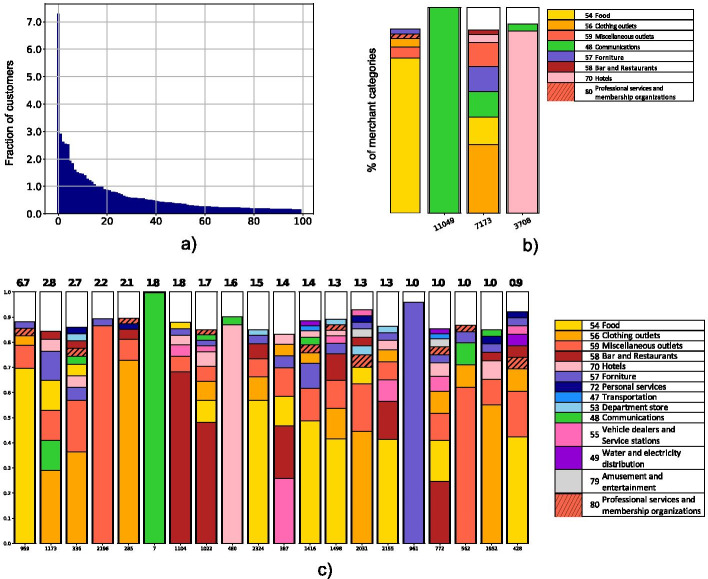
In **a** the distribution of the size of the consumption profiles—fraction of customers belonging to a consumption profile over the customer population. In the figure we report the top 100 consumption profiles ordered by their size. In **b** a selection of representative consumption profiles, visualized as stacked barplots. In **c** the 20 most frequent representative consumption profiles extracted from the similarity graph on the entire dataset. The profiles are ordered by the fraction of adoption displayed on top of each stacked bar. In **b**, **c** we display only categories associated with a component of the representative consumption profile greater than 0.01, for the sake of readability

### Customer and representative consumption profiles

The above distributions have highlighted a certain level of heterogeneity in which merchant macro-categories are targeted by clients, and in the number of macro-categories customers have spent their money in. This observation is supported by the distribution of the cosine similarity among each pair of customer consumption profiles. In fact, more than 60% of pairs have zero-similarity and about 2% of pairs have a similarity greater than 0.8, a medium-high similarity. In Fig. 3c we show some examples which capture the diversity in the consumption behaviors in terms of available money allocation. A’s consumption profile stands for a typical daily purchase behavior, because it spends almost 90% of its available capacity in essential goods like food (macro-category 54—Grocery). B distributes its purchases in a very different way: first, the distribution is more heterogeneous; second, the food category is not the most important one. As the last example, C has a completely different spending pattern, where the macro-category 54 is not present at all; this suggests a very specific use of the credit card typical of a professional activity.

After computing the consumption profile for all clients, we build the similarity graph. Specifically, here we show the results by taking into account edges (*u*, *v*) with $sim(u,v)>0.98$, but we have also conducted a sensitivity analysis on the threshold by varying $\Delta$ in the interval [0.8, 0.98]^3^ with a 0.05 spacing. We evaluated the similarity between each pair of thresholds by the normalized mutual information (NMI), a standard method for the evaluation of the accordance between partitions resulting from community detection algorithms. We found an average NMI equal to 0.98, which indicates a high level of accordance for each pair of thresholds, and, in general, it suggests a low impact of the threshold on the results, in the range [0.8, 0.98].

The threshold 0.98 led to a graph with 229,951 nodes and more than 100 million links, on which we apply the community detection algorithm to catch the groups of clients with similar spending behavior and to identify representative consumption profiles. The distribution of the customers among the communities/consumption profiles is reported in Fig. 4a, where each bar represents the fraction of customers belonging to a specific community over the number of clients in the similarity graph, and communities are sorted by that fraction. It is evident how the heterogeneity impacts the distribution of the customers, in fact, we observe a primary group containing more than $7\%$ of customers, followed by many smaller communities. Since the outcomes may be influenced by the choice of the community detection algorithms, we compare the partitions returned by Louvain with partitions returned by the Infomap method (Rosvall et al. 2009). Unlike Louvain, Infomap is not a modularity optimization algorithm, rather it adopts a different strategy based on the behavior of a random walker. In evaluating the accordance between the two strategies, we measure the normalized mutual information between the partitions returned by the two algorithms varying the similarity threshold, as in the threshold sensitivity analysis. In all the cases, we got NMI scores between 0.96 and 0.99. So, the choice of the community detection algorithm has a low impact on the results, since the similarity graph has a highly modular structure, as highlighted by the modularity scores greater than 0.75 obtained by both Louvain and Infomap algorithms for each similarity threshold.

For each community, we compute its centroid, i.e. its representative consumption profile, that represents the average behavior of clients in a community. Through a visualization with stacked barplot, we immediately catch the differences: an example is shown in Fig. 4b. Here, we report four different types of representative profiles, showing how the clients’ consumption behaviors are heterogeneous. For instance, community 6401 is mainly populated by clients that present a typical spending behavior, focused on food and with a small percentage of other types of purchases. On the contrary, community 11049 is really particular, because, on average, its clients use their credit card for communication purchases only (probably for phone cards). Another example of centroid that highlights a well defined type of purchases is community 2049, where clients spend mostly in hotels. Finally, the whole set of representative consumption profiles also contains well diversified consumption behaviors, such as community 3237 as well as exclusive usage like the previous communities. In general, the heterogeneity of the spending behaviors is depicted in Fig. 4c, where we report the 20 most frequent representative consumption profiles on the whole set of customers, ordered by the percentage of usage.

### Consumption in urban areas

We analyze the cities from different viewpoints. Initially, we study how much clients coming from different areas spend in different macro merchant categories; secondly, we apply the centroid method to a graph composed only by customers that live in one of the selected cities. The selection was made by considering the five most populated urban areas: Milan, Brescia, Bergamo, Turin and Genoa. In the last part of this work, we use a rank correlation approach in order to evaluate the similarity between cities.

#### Amount distribution

Before delving into how citizens of different cities distribute their spending amount among merchant categories, we focus on the spending capacity in the cities, i.e. how much money people spend in the merchant categories. In order to compare the amounts of money spent in different categories in different cities, we consider the set of not normalized vector ${\mathbf {v}}^c$ introduced in “Customer representation through consumption profile" section. Specifically, for each merchant category *m*, we inspect the distribution of the average monthly purchases in category *m*, over the customers residing in a city. The resulting distributions are entirely reported in the Support Information; here we comment on the most significant categories in terms of common and/or specific behaviors. A descriptive summary of the selected distributions in terms of percentiles have been reported in Table 2. Category 48 (Communications) is an example of common behavior because the values are very similar among cities. In fact, the purchases of this category are related to monthly phone or Internet rates, which generally do not depend on the geographic position but are nation-wide. On the other hand, other categories exhibit a discernible feature for some cities. For example, in category 56 (Clothing), the citizens of Genoa spend less with respect to the other cities, especially Milan. The city of Milan and Genoa are also pretty different in a further category: 70 (Hotels). Here, the 75*th* percentile of the monthly amount spent by the citizens of Milan is over 63 while for Genoa citizens is about 40. Speaking about Cinema services—78-, Milan and Brescia have comparable distributions while Bergamo follows a specific distribution different from the other ones; Bergamo citizens spend more on this category. On the other hand, Turin has in general lower values. Finally, we observe a quite common trend for category 54 (Food); the five cities maintain the same order of magnitude for the whole distribution; even if citizens of Bergamo spend the most in food.

**Table 2:** Average spending amount

City	Percentile			
	25	50	75	95
*54-Food*				
Milan	2	7	34	234
Brescia	2	7	30	210
Bergamo	2	8	38	283
Turin	2	6	24	166
Genoa	1	5	22	151
*56-Clothing*				
Milan	2	6	18	82
Brescia	2	6	17	81
Bergamo	2	6	17	80
Turin	2	6	18	75
Genoa	2	5	13	53
*70-Hotels*				
Milan	7	24	63	211
Brescia	6	20	50	171
Bergamo	7	21	55	181
Turin	6	18	48	159
Genoa	5	16	41	127
*78-Cinema services*				
Milan	1	2	6	26
Brescia	1	2	6	25
Bergamo	1	3	8	27
Turin	1	2	5	19
Genoa	1	2	5	25
*48-Communications*				
Milan	2	6	18	62
Brescia	2	6	18	53
Bergamo	2	6	17	53
Turin	2	6	19	62
Genoa	2	6	19	60

Summary of the distribution of the average monthly spending amount in the five selected merchant categories: food, clothing, hotels, cinema services and communications. For each distribution in each city, we report the 25*th*, 50*th*, 75*th* and 95*th* percentile

#### City consumption profiles

The results about the monthly average amount grouped by merchant categories have highlighted differences in the spending behavior of the cities; however, it is difficult to decouple the cost of living in a city and the citizens’ real attitude in spending. The definition of consumer profile reduces the effect of the cost of living since it expresses how people allocate their money; while we highlight the behavioral aspects of citizens by means of a graph-based approach. In fact, we apply all the operations explained in “City consumption profile” section to the set of clients living in the chosen cities—43, 906 customers. So, each city is characterized by a proper consumption profile, i.e. a sequence of spending behaviors ordered by the frequency of usage.

The city consumption profiles have been reported in Fig. 6 in the “Appendix”. Specifically, the profiles have been limited to the 20 most frequent representative profiles. A first visual inspection of the city profiles tells a lot about the behavior of their citizens. The first observation is that there is a representative consumption profile, common to the five cities and nearly always at the first position in the city consumption profiles. In this profile—ID 959 in the barplots of Fig. 6—the main merchant category is Food followed by goods sold in miscellaneous outlets. This is a widespread spending behavior where the credit card is the main mean for purchasing of primary goods. In terms of frequency of usage, this profile is more spread in Bergamo and Brescia than in Genoa and Turin, where the highest ranked consumption profiles are less frequent than in the remaining cities. A further common aspect to four out of five cities is the rank associated to the consumption profile almost exclusively characterized by miscellaneous outlets—from 4*th* to 6*th* position. In all cities, this consumption profile is adopted by on average $1\%$ of citizens, who use their credit card for purchases in different outlets belonging to a single generic merchant category. A similar observation about the ranking characterizes the consumption profile related to Clothing outlets. It is quite important in all the cities, but it has a larger variability of the fraction of citizens adopting it, when comparing it with the previous consumption profile (Miscellaneous outlets). It ranges from 0.7 to $1.4\%$ of citizens. The main differences among the city consumption profiles are due to a few consumption profiles having a dominant merchant category, i.e. a specialized usage of the credit card for a single type of purchase. The Communications (green bar), the Hotels (pink) and the Forniture (purple) profiles are of this kind. In particular, the Communications profile is very popular— rank 3—and spread—$1.5\%$—in Brescia, but less important (ranking) and frequent (percentage of adoption) in the remaining cities. A similar observation holds for the Hotels profile, which is among the top 10 consumption profiles in Bergamo, Brescia and Genoa, but less important and spread in Turin and Milan.

**Fig. 5:**
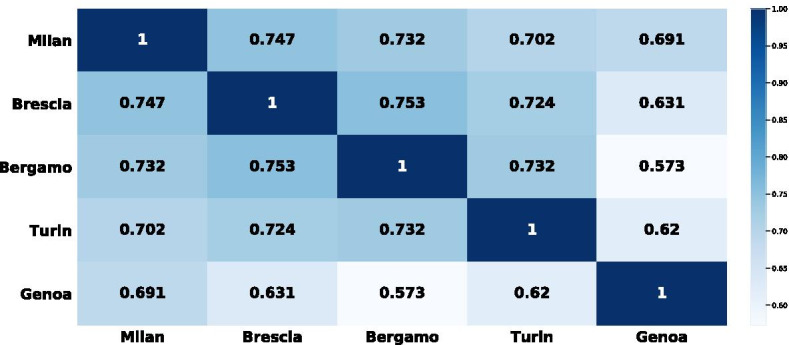
Matrix of the city consumption similarity index. Rows and columns report the five selected city. Each element of the matrix denotes the weighted Kendall’s coefficient computed on the rank induced by the city consumption profile

**Fig. 6:**
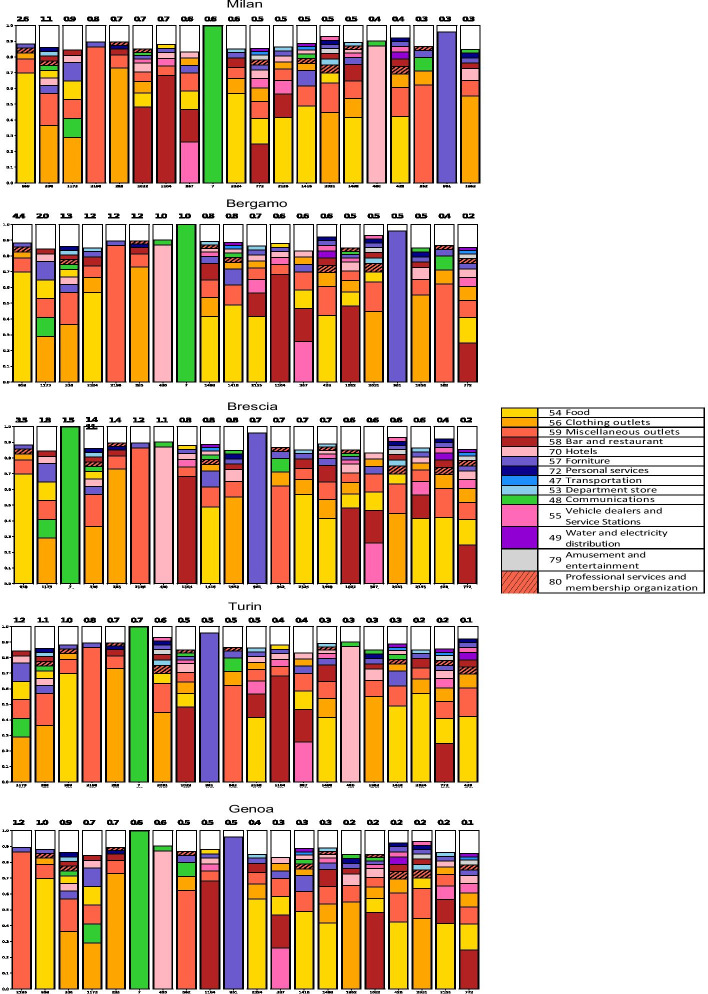
City consumption profiles for the five selected cities: Milan, Bergamo, Brescia, Turin and Genoa

We can also exploit the city consumption profiles to provide a quantitative measure of how similar two cities are in terms of adoption of spending behaviors. To this aim, in “City consumption profile” section we defined the city consumption similarity index, an index which returns the overall similarity between two cities based on their city consumption profile. In Fig. 5 we report a similarity matrix among the five cities, where an element $(c_i,ci_j)$ denotes the city consumption similarity index between city $c_i$ and city $c_j$. Bergamo and Brescia are the most similar cities in terms of city consumption profiles (0.93), while Bergamo and Genoa get the lowest similarity index (0.573). In fact, Genoa has a city consumption profile slightly different from the remaining cities, as we also observed along the analysis of the stacked barplots in Fig. 6. On the contrary, the remaining cities form a group characterized by a quite similar adoption of consumption profiles.

In general, the above analyses and comparisons of the city consumption profiles highlight that:

- there is a set of most important consumption profiles common to all the cities, but what differentiates the cities is the fraction of citizens who adopt a specific spending behavior. That impacts the frequency of adoption of the consumption profiles and their relevance. In particular, the rank of some mono-categorical consumption profiles depends on the city, supporting the observations that each city and its citizens has specific behaviors in managing and allocating some kind of purchases;
- although city diversity depends on some consumption profiles, we observe that part of the overall city profiles are common to all cities; in particular, a fraction of citizens uses credit card payments for primary goods, such as food and clothing;
- there is a subset of cities whose citizens behave quite similarly in terms of purchase allocation among the merchant categories and usage behavior of the credit card, while the city of Genoa is characterized by a bit different general behavior. This is due to the adoption of spending behavior related to the primary goods, in fact in Genoa the highest ranked consumption profile is related to clothing and not to food.

## Conclusion

Human behavior has been one of the most important, interesting, and attractive topics since a long time ago; from the microscopic level related to the behavioral profile of a single person to the mesoscopic one of groups of people, to the macroscopic profiling of small or large populations. The explosion of digital data since the beginning of this millennium has given a turning point to these studies both from a theoretical and an applicative point of view. From a theoretical point of view, the typical studies in the humanities have been flanked by researches based on data mining, network science and machine learning techniques. On the other side, more and more industrial and applicative fields are leveraging people’s profiles to enhance the services they offer. Also, economic and financial institutions such as banks are moving toward this paradigm, not only to provide better services to their clients, but also to perform risk assessment analysis, both by embedding behavioral data (Zand 2020). This piece of work takes its beginning from customers’ credit cards to build a behavioral customer segmentation based on their purchasing profile. But in this paper, customer segmentation based on their spending behavior is declined within the field of urban science. In fact, the application to the macroscopic level of city populations offers a methodology for the construction of city consumption profiles. The proposed network-based methodology is completely general and applicable in any context of cashless payments. The case study, relating to the consumption of goods via credit cards only, reveals interesting patterns of the selected cities. Overall, results show that beyond primary goods and services consumption, each city is unique and cities which might seem similar, actually exhibit different profiles.

## Figures and Tables

**Fig. 7:**
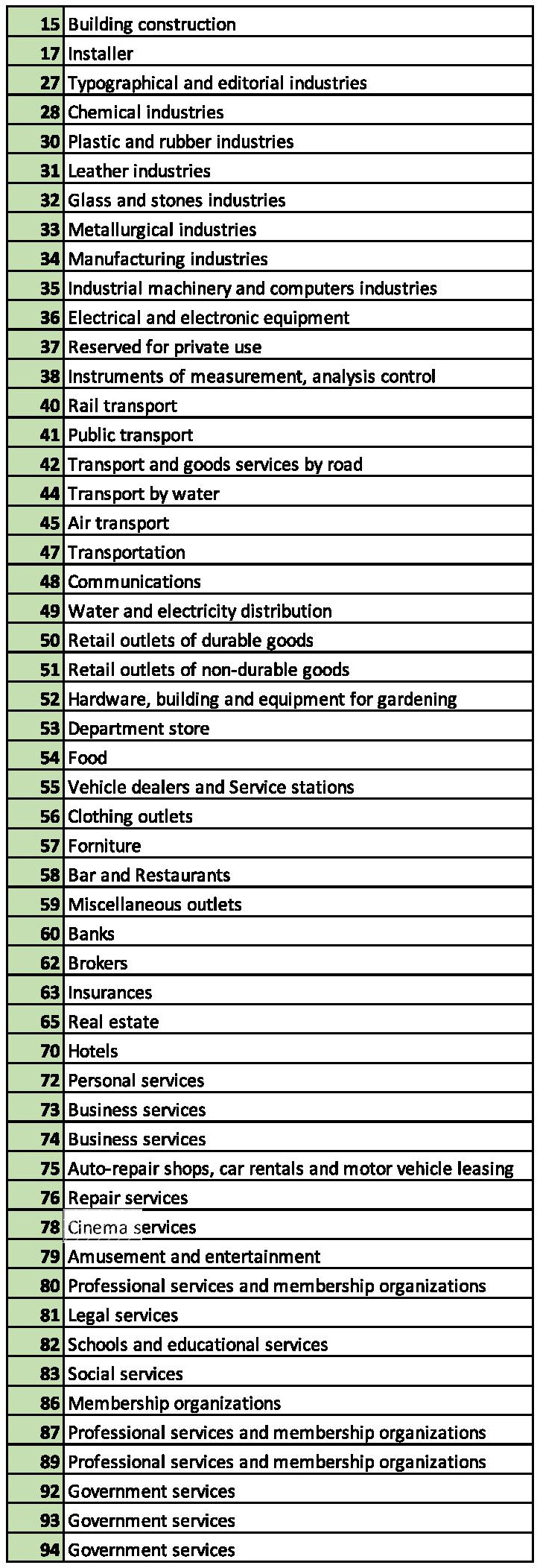
Table of all MCC descriptions

## Data Availability

The data that support the findings of this study are available from an Italian financial group but restrictions apply to the availability of these data, and so are not publicly available.

## Appendix

See Figs. 6 and 7.

## Abbreviations

MCC: Merchant Category Code
CDF: Cumulative distribution function
NMI: Normalized mutual information

## Footnotes

1 For completeness, we also run the entire pipeline for the identification of groups of similar customers by replacing the tail cut with a feature selection method based on the variance for an unsupervised setting (Solorio-Fernández et al. 2020). We evaluated the accordance of the final partitions by the normalized mutual information for different values of the similarity threshold. In all the cases, we got NMI values between 0.49 and 0.67, medium-high values which suggest a good level of accordance between partitions.

2 The original merchant code system is available at https://www.tuttocamere.it/files/attivita/Codici_SIC.pdf in Italian. In the table we translate and report the macro-categories only.

3 For values less than 0.8, we experienced scalability issues due to the number of links of the similarity graph.

## References

[CR1] Alshamsi A, Awad E, Almehrezi M, Babushkin V, Chang P-J, Shoroye Z, Tóth A-P, Rahwan I (2015). Misery loves company: happiness and communication in the city. EPJ Data Sci.

[CR2] Batty M (2021). Defining urban science.

[CR3] Blondel VD, Guillaume J-L, Lambiotte R, Lefebvre E (2008). Fast unfolding of communities in large networks. J Stat Mech Theory Experiment.

[CR4] Calabrese F, Ferrari L, Blondel VD (2014). Urban sensing using mobile phone network data: a survey of research. ACM Comput Surv.

[CR5] Chong SK, Bahrami M, Chen H, Balcisoy S, Bozkaya B (2020). Economic outcomes predicted by diversity in cities. EPJ Data Sci.

[CR6] Citybank: Merchant Category Codes. https://www.citibank.com/tts/solutions/commercial-cards/assets/docs/govt/Merchant-Category-Codes.pdf

[CR7] Daniele Q, Schifanella R, Aiello LM et al (2014) The shortest path to happiness: recommending beautiful, quiet, and happy routes in the city. In: 25th ACM conference on hypertext and social media. ACM-Association for Computing Machinery, pp 116–125

[CR8] De Nadai M, Xu Y, Letouzé E, González MC, Lepri B (2020). Socio-economic, built environment, and mobility conditions associated with crime: a study of multiple cities. Sci Rep.

[CR9] Di Clemente R, Luengo-Oroz M, Travizano M, Xu S, Vaitla B, González MC (2018). Sequences of purchases in credit card data reveal lifestyles in urban populations. Nat Commun.

[CR10] Kitchin R, Lauriault TP, McArdle G (2015). Knowing and governing cities through urban indicators, city benchmarking and real-time dashboards. Reg Stud Reg Sci.

[CR11] Leo Y, Karsai M, Sarraute C, Fleury E (2018). Correlations and dynamics of consumption patterns in social-economic networks. Soc Netw Anal Min.

[CR12] Lobo J, Alberti M, Allen-Dumas M, Arcaute E, Barthelemy M, Bojorquez Tapia LA, Brail S, Bettencourt L, Beukes A, Chen W-Q et al (2020) Urban science: integrated theory from the first cities to sustainable metropolises

[CR13] Mihova V, Pavlov V (2018) A customer segmentation approach in commercial banks. In: AIP conference proceedings, vol 2025. AIP Publishing LLC, p 030003

[CR15] Moustaka V, Vakali A, Anthopoulos LG (2017) Citydna: smart city dimensions’ correlations for identifying urban profile. In: Proceedings of the 26th international conference on world wide web companion, pp 1167–1172

[CR14] Moustaka V, Maitis A, Vakali A, Anthopoulos LG (2020). Citydna dynamics: a model for smart city maturity and performance benchmarking. Companion Proc Web Conf.

[CR16] Ratti C (2018) 11. senseable city. Dialogues on Design: Notes on Doctoral Research in Design 2018, p. 149

[CR17] Rosvall M, Axelsson D, Bergstrom CT (2009). The map equation. Eur Phys J Spec Top.

[CR18] Silva TH, Viana AC, Benevenuto F, Villas L, Salles J, Loureiro A, Quercia D (2019). Urban computing leveraging location-based social network data: a survey. ACM Comput Surv (CSUR).

[CR19] Solorio-Fernández S, Carrasco-Ochoa JA, Martínez-Trinidad JF (2020). A review of unsupervised feature selection methods. Artif Intell Rev.

[CR20] United Nations UN (2021) The value of sustainable urbanization. https://unhabitat.org/sites/default/files/2020/10/wcr_2020_report.pdf

[CR21] Vigna S (2015) A weighted correlation index for rankings with ties. In: Proceedings of the 24th international conference on world wide web. International World Wide Web Conferences Steering Committee, pp. 1166–1176. 10.1145/2736277.2741088

[CR22] Xiaowen D, Eaman J, Alfredo JM, Burçin B, Bruno L, Alex Sandy P (2020). Purchase patterns, socioeconomic status, and political inclination. World Bank Econ Rev.

[CR23] Zakrzewska D, Murlewski J (2005) Clustering algorithms for bank customer segmentation. In: 5th International conference on intelligent systems design and applications (ISDA’05). IEEE, pp 197–202

[CR24] Zand SK (2020) Towards intelligent risk-based customer segmentation in banking. arXiv:2009.13929

[CR25] Zheng Y, Capra L, Wolfson O, Yang H (2014). Urban computing: concepts, methodologies, and applications. ACM Trans Intell Syst Technol (TIST).

[CR26] Zygiaris S (2013). Smart city reference model: assisting planners to conceptualize the building of smart city innovation ecosystems. J Knowl Econ.

